# Development of practice guidelines for daily oral care in care‐dependent older adults to complement the InterRAI suite of instruments using a modified Delphi approach

**DOI:** 10.1111/opn.12351

**Published:** 2020-10-19

**Authors:** Stefanie Krausch‐Hofmann, Ellen Palmers, Dominique Declerck, Joke Duyck

**Affiliations:** ^1^ Department of Oral Health Sciences, Population Studies in Oral Health KU Leuven Leuven Belgium; ^2^ Department of Oral Health Sciences, Biomaterials/BIOMAT KU Leuven Leuven Belgium

**Keywords:** care of older people, nursing care, oral health, oral hygiene

## Abstract

**Aim:**

To develop practice guidelines for nursing assistants who provide daily oral care to older adults.

**Background:**

The interRAI suite of instruments is internationally used in professional health care to assess the needs of care‐dependent older persons. An optimised oral health section was developed recently to identify care clients with poor oral health and hygiene. Internationally shared guidelines for daily oral care are needed to complement the optimised oral health section of the interRAI suite of instruments.

**Material and methods:**

The modified Delphi approach started with the preparation of an initial draft. Subsequently, an online survey and a face‐to‐face discussion were conducted with international experts. Their feedback was used to revise the draft. Two additional online surveys were conducted with the experts to reach consensus agreement for each item of the revised version. The same group of experts was invited to the different study phases.

**Results:**

The three surveys were completed by 26, 27 and 23 international experts, respectively. A group of 18 experts completed each survey, whereof a subgroup of 11 experts also took part in the face‐to‐face discussion. Experts were dental hygienists, dentists, nursing scientists, physicians and psychologists from 14 different countries. After the final survey, consensus agreement was reached for 54 of the 57 (94.7%) items, representing the final version of the guidelines.

**Conclusion:**

Available evidence was combined with practical feedback from international experts to develop clear and concise practice guidelines for daily oral care in older adults.

**Implications for practice:**

The guidelines will help to improve knowledge and reduce barriers of nursing assistants to provide daily oral care.

**Summary statement of implications for practice**

1 **What does this research add to existing knowledge in gerontology?**

- Clear and concise practice guidelines for daily oral care in care‐dependent older adults were developed.
- The available evidence was complemented with practice‐based feedback and consensus agreement from international experts.
- Contradicting national guidelines were harmonised.

2 **What are the implications of this new knowledge for nursing care with older people?**

- The guidelines will help to improve knowledge and reduce barriers of nursing assistants to provide daily oral care.
- By facilitating adequate daily oral care, oral health will improve. In turn, this will contribute to better general health and quality of life.

3 **How could the findings be used to influence policy or practice or research or education?**

- The guidelines complement the optimised oral health section that recently was developed for the interRAI suite of instruments.
- The guidelines are available for all settings where daily oral care is provided to care‐dependent older adults.
- The guidelines are also applicable for education and training of nursing assistants or other direct care providers.

## 1 INTRODUCTION

Human lifespan is increasing in all regions of the world (Roser et al., 2019). Considering individual well‐being and health system sustainability, optimal health is preferred for these added years. One of the key factors of healthy ageing is good oral health. The latter is associated with general health and with quality of life (Bidinotto et al., 2016; Dietrich et al., 2017; Graziani et al., 2018; Iwasaki et al., 2018; Rouxel et al., 2018; Teeuw et al., 2014; Teixeira et al., 2014; Tran et al., 2018). Over the last three decades, older adults in high‐income countries keep their natural teeth longer and prevalence rates of edentulism are declining (Müller et al., 2017; Tyrovolas et al., 2016). Another trend is the increased use of oral implants to replace missing teeth (Elani et al., 2018). As a result, more care‐dependent older adults have at least some natural teeth or complex dental restorations.

An effective daily oral care is indispensable to maintain good oral health. It mainly aims to prevent dental decay and gum disease by disintegration of the bacterial biofilm and application of active ingredients (Sbordone & Bortolaia, 2003). In care‐dependent older adults, preventive measures are particularly relevant as curative treatment is often challenged by cognitive and physical impairment. In addition, evidence is available that daily oral care reduces the risk of aspiration pneumonia (Sjögren et al., 2016; Van Der Maarel‐Wierink et al., 2013).

Older persons in professional care settings often lack the ability to perform adequate daily oral care independently. This task is usually delegated to nursing assistants who are also called nurse's aides or health care aides (Hoben et al., 2017).

Studies consistently show that oral hygiene in care‐dependent older adults is deficient (De Visschere et al., 2016; Delwel et al., 2018; Yoon et al., 2018). This indicates the existence of barriers that prevent nursing assistants from providing proper daily oral care (Göstemeyer et al., 2019; Hoben et al., 2017). Adequate daily oral care requires skills to assess individual needs and to select and apply devices correctly. In addition, effective strategies are needed to cope with physical impairment and care‐resistant behaviour. However, oral health‐related knowledge and training are often insufficient in nursing assistants (Catteau et al., 2016; Mehl et al., 2016; Wårdh et al., 2012). This constitutes one of the main barriers to provide daily oral care (Göstemeyer et al., 2019; Hoben et al., 2017).

One component to facilitate daily oral care is the availability of clear and concise practice guidelines for nursing assistants. Scientific evidence is scarce with respect to oral care devices and approaches for older care‐dependent adults. At the same time, numerous national guidelines are available that include heterogeneous or contradictory advice. While US guidelines indicate to rinse with water after brushing (National Institute of Dental & Craniofacial Research – U.S. National Institutes of Health, 2018), guidelines from the UK advise to only spit out toothpaste (Public Health England ‐ Department of Health, 2017). A further example relates to overnight storage of removable dentures. Guidelines from the Netherlands advise dry storage (The Netherlands ‐ Beroepsvereniging van Verpleeghuisartsen en Sociaal Geriaters, 2007), but Australian and US guidelines recommend storage under wet conditions (Centre for Oral Health Strategy – Australia New South Wales Ministry of Health, 2014; National Institute of Dental & Craniofacial Research – U.S. National Institutes of Health, 2018). These discrepancies preclude international application of the available guidelines.

The interRAI suite of instruments is used in 35 countries to assess the needs and capacities of care‐dependent individuals (InterRAI, 2020). For its oral health section, internationally acknowledged guidelines for daily oral care are needed.

The first version of the interRAI assessment instrument became known as ‘Minimum Data Set’. It was developed during the 1980 s in the United States to improve the identification of care needs in nursing home residents. The items register physical, cognitive, psychometric and sociographic strengths and needs of a person. In the following years, the interRAI research network was established and versions for other care settings were developed. For example, home care and palliative care versions became available. To harmonise these different versions, the interRAI suite of instruments was released in 2005. It consists of a core of common items that is complemented by sector‐specific items. All versions share a consistent terminology and apply the same registration methods. Based on the assessment, multiple outcome measures are calculated to assist care planning. While the scales allow an instant overview of the condition of a person, Clinical Assessment Protocols indicate specific risks and resources. Periodical use of the interRAI instruments allows to detect changes of the client and to adapt care planning. To further improve the interRAI system, its components are evaluated and updated on a regular basis (Vanneste & Declercq, 2014).

An oral health section is included in the interRAI versions for home care and for long‐term care facilities. Both versions are mainly used in care for older adults. An optimised version of this oral health section was recently developed. One of its aims is the identification of clients with non‐acceptable oral hygiene (Krausch‐Hofmann et al., 2020). To complement this optimised oral health interRAI section, practice guidelines for daily oral care were developed in the present study. A modified Delphi approach was used to combine the available evidence with practice‐based feedback and consensus from international experts.

## 2 METHODS

### 2.1 Preparation phase

**Figure 1:**
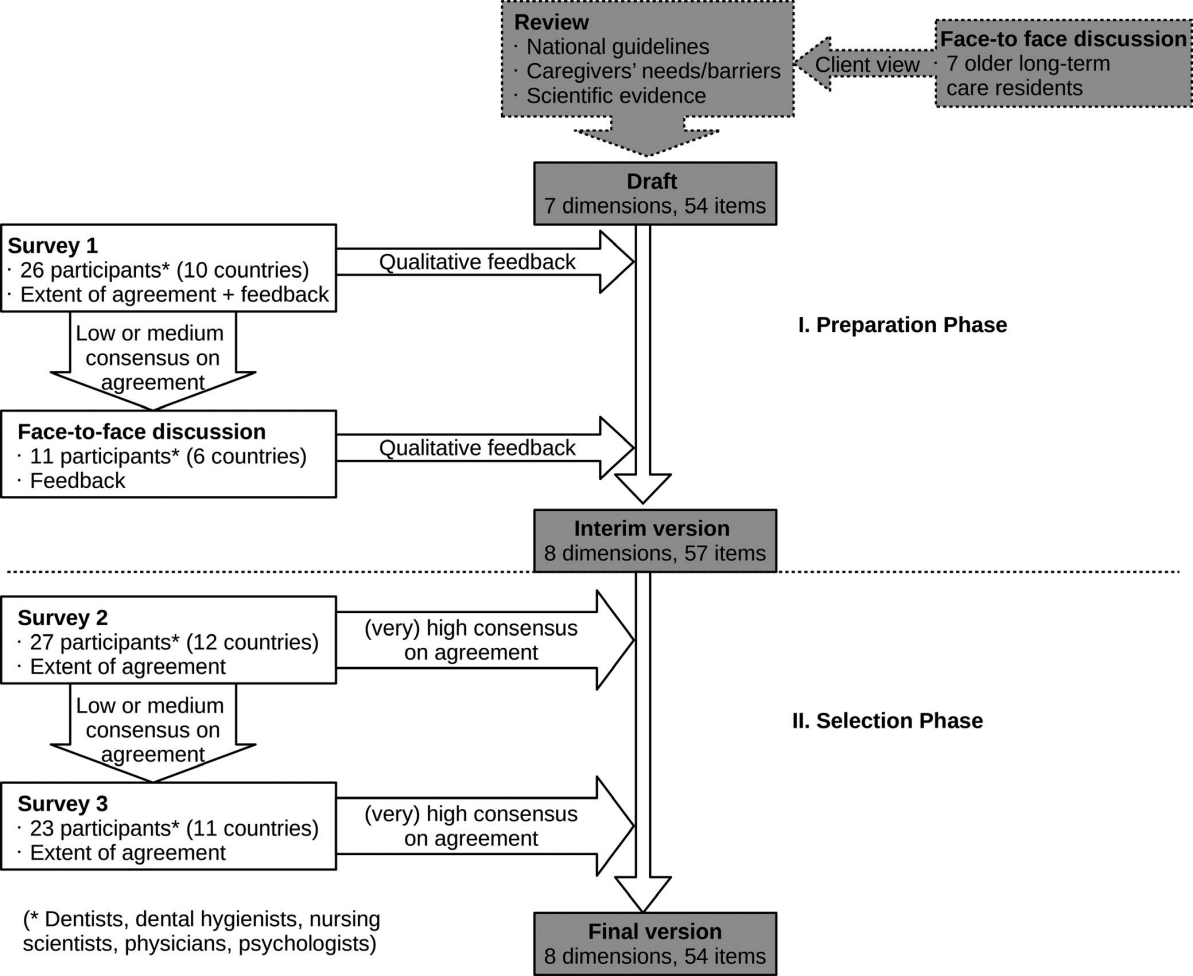
Overview of the study phases.

Figure 1 provides an overview of the different study phases.

An initial draft of the guidelines was based on a review of existing guidelines on preventive oral care published by national health ministries or professional health associations from four different countries. The countries were located at three different continents where instruments of the interRAI suite are used (Centre for Oral Health Strategy – Australia New South Wales Ministry of Health, 2014; National Institute of Dental & Craniofacial Research – U.S. National Institutes of Health, 2018; Public Health England ‐ Department of Health, 2017; The Netherlands ‐ Beroepsvereniging van Verpleeghuisartsen en Sociaal Geriaters, 2007). Only the chapters on daily oral care were considered. Items were formulated for different domains such as *Care for Natural Teeth* or *Approaches for Clients with Care*‐*Resistant Behaviour*. The items advised concrete devices, techniques or strategies. Reference lists of the reviewed national guidelines and additional literature were searched. The results of this search were used to formulate further items and to indicate the scientific evidence of each item in the draft. A face‐to‐face discussion was held with a group of seven older long‐term care residents to incorporate the view of this group.

The initial draft of the guidelines was compiled by the research team of this study. The group consisted of three dentists with academic‐clinical expertise in geriatric and special needs dentistry and one health care psychologist.

To recruit international experts, a call was circulated among members of the *European College of Gerodontology*. In addition, emails were sent to dental hygienists, dentists, nursing scientists, physicians and psychologists with an academic‐clinical background related to oral health in care‐dependent older adults. The experts had to be affiliated with an organisation located in a country where instruments of the interRAI suite were used.

The two following steps of the preparation phase aimed to revise the initial draft based on feedback from the international experts. An online survey (*survey 1*) and a face‐to‐face discussion were conducted.

In *survey 1*, quantitative and qualitative feedback was provided by the experts. Participants indicated the extent of agreement with each item on a 5‐point Likert scale (1 strongly agree, 2 agree, 3 nor agree/nor disagree, 4 disagree, 5 strongly disagree). They were instructed to assume that resources in terms of staffing, time and material were sufficiently available. Participants were invited to provide general and item‐wise feedback and to suggest additional items.

The face‐to‐face discussion was conducted with a subgroup of experts during a conference meeting of the *European College of Gerodontology*. Items that reached only medium or low consensus on agreement in *survey 1* were considered. The experts discussed how these items could be revised or whether they should be eliminated. Participants were further invited to suggest additional domains or items. The face‐to‐face discussion was moderated by one of the researchers (SKH). A co‐moderator (EP) took notes of the discussion.

All qualitative feedback contributions provided by the experts in *survey 1* and during the face‐to‐face discussion were appraised by the research team and used to revise the draft. This resulted in an interim version of the guidelines.

### 2.2 Selection phase

The selection phase consisted of two online surveys (*survey 2* and *survey 3*). It aimed to select those items from the interim version that reached high or very high consensus on agreement among the international experts. The same experts were invited as in the preparation phase. Both surveys collected quantitative feedback, using the 5‐point Likert scale described above. Consensus on agreement was defined by three measures: percentage of the sum of the responses ‘strongly agree’ and ‘agree’, the median and the Inter Quartile Range (IQR) (Table 1) (Jünger et al., 2012).

In *survey 2*, participants assessed each item of the interim version. Items that did not achieve high or very high consensus on agreement in *survey 2* were re‐assessed in *survey 3*. In this last survey, experts were provided with a summary of the scientific evidence and the results of the previous steps for each item. This approach is a characteristic of Delphi studies and allows participants to reconsider their responses.

**Table 1:** Consensus agreement, based on Jünger et al. (2012)

Consensus on agreement	Percentage of agreement (strongly agree +agree), in %	Median	IQR
Very high	≥80	1	0
High	≥80	≤2	1
Moderate	60–79	≤2	1
Low	<60	>2	>1

The final version of the guidelines was constituted by those items that achieved high or very high consensus on agreement in *survey 3* at the latest.

Quantitative statistical analyses were performed with SPSS version 23.

### 2.3 Ethics

Following the Belgian law (Wet inzake experimenten op de menselijke persoon, 2004), approval from a medical ethics committee was not necessary for this study. Data were collected anonymously, and participation was completely voluntarily. Participants provided consent that data would be used for education and research purposes.

## 3 RESULTS

Table 2 shows the characteristics of the study participants. A group of 18 experts completed all surveys, whereof a subgroup of 11 experts also took part in the face‐to‐face discussion. Table 3 provides an overview of the results for each survey.

**Table 2:** Characteristics of the international experts

Phase	Step	N	Professional background (number of participants)	Country (number of participants)
Preparation	Draft	3	Dentist (3), psychologist (1)	Belgium (4)
	Survey 1	26	Dental hygienist (4), dentist (19), nursing scientist (1), physician (1), psychologist (1)	Belgium (5), Canada (1), Chile (1), Hong Kong (1), Malta (4), Poland (1), Sweden (2), The Netherlands (5), UK (1), USA (5)
	Face‐to‐face discussion	11	Dental hygienist (3), dentist (7), nursing scientist (1)	Belgium (2), Chile (1), 2 Malta (2), Poland (1), The Netherlands (3), USA (2)
Selection	Survey 2	27	Dental hygienist (3), dentist (18), nursing scientist (1), physician (3), psychologist (1), other (1)	Belgium (5), Canada (1), Chile (1), Finland (1), Hong Kong (1), Malta (2), New Zealand (1), Poland (1), Sweden (2), Switzerland (1), The Netherlands (6), USA (5)
	Survey 3	23	Dental hygienist (1), dentist (18), nursing scientist (1), hysician (2), psychologist (1)	Belgium (5), Canada (1), Chile (1), Germany (1), Malta (2), New Zealand (1), Poland (1), Sweden (2), The Netherlands (5), UK (1), USA (3)

18 participants completed all surveys, and a subgroup (*n* = 11) also took part in the face‐to face discussion.

**Table 3:** Consensus on agreement (CA) in the three survey rounds for each item of the guidelines

	Preparation phase		Selection phase	
	CA survey 1	Changes	CA survey 2	CA survey 3
Care for natural teeth				
Toothpaste				
With fluoride	High	Adjusted	Very high	‐
Sodium Lauryl Sulfate‐free	‐	Additional	High	‐
Amount in proportion with number of teeth	Low	Removed	‐	‐
Toothbrush				
Manual or powered	High	Adjusted	High	‐
Size toothbrush head	High	Merged	High	‐
Bristle stiffness	Low			
Adaptive aids	‐	Additional	High	‐
Procedures				
Timing and frequency of oral care	High	No change	High	‐
Brushing technique	High	No change	Very high	‐
Post‐brushing rinsing	High	Adjusted	High	‐
Interdental cleaning				
Frequency	High	Adjusted and merged	High	‐
Devices	Low			
Additional fluoride				
High‐fluoride toothpaste	High	Adjusted and merged	Very high	‐
Fluoride mouth rinse	Low			
Chlorhexidine				
Indication and methods of application	Low	Adjusted	Moderate	Moderate
Care for full or partial removable dentures				
Mechanical cleaning				
Non‐abrasive denture cleanser	High	No change	High	‐
Denture brush	High	Adjusted	Very high	‐
Chemical cleaning				
Soaking in denture‐cleansing solution	Low	Adjusted	Low	Low
Procedures				
Rinsing with water after meals	High	No change	High	‐
Breaking protection	High	No change	High	‐
Overnight storage				
Remove from mouth	High	No change	High	‐
Dry storage	Low	Adjusted	High	‐
Denture‐related oral care				
Denture retainers	Very high	No change	High	‐
Connection bar	Very high	No change	High	‐
Denture adhesive	‐	Additional	High	‐
Care for the tongue				
Device				
Loop‐shaped tongue cleaner	Low	Adjusted	High	‐
Procedures				
Indication and frequency	High	Adjusted	High	‐
Cleaning technique	High	Adjusted	High	‐
Maintenance of oral care utensils				
Daily maintenance				
Daily maintenance of brushes	High	Adjusted	High	‐
Replacement				
Replacement of brushes	High	Adjusted	High	‐
Approaches for clients who need guidance or help				
Reminders and triggers				
Pictures in the bathroom	‐	Additional	High	‐
Oral care in front of the sink	High	Item split	High	‐
Oral care devices			High	‐
Hands‐on assistance				
Caregiver guides the toothbrush	High	Adjusted	High	‐
Caregiver finalises oral care	‐	Additional	High	‐
Caregiver provides oral care				
Position of the caregiver	High	Removed	‐	‐
Position of the client	High	Adjusted	High	‐
Devices to keep the mouth open	High	Adjusted	Moderate	High
Tell‐show‐do approach	Very high	No change	High	‐
Removal of fluids	‐	Additional	High	‐
Approaches for care‐resistant behaviour				
Cognitively competent clients				
Informed decision	High	No change	High	‐
Respect for persistent refusal	Low	Adjusted	High	‐
Situation				
Dental care routine	High	No change	Very high	‐
Quiet environment	High	No change	Very high	‐
Approach				
At eye level	High	Adjusted	Very high	‐
Physical contact	Very high	No change	High	‐
Smiling and humour	High	Adjusted	High	‐
Distract client by talking	High	Merged	High	‐
Distract client by providing objects	High			
Client initiates or completes oral care	High	Merged	High	‐
Caregiver guides brushing movements	High			
Client observes oral care in the mirror	Low	Adjusted	Low	Low
Second caregiver takes over	Low	Adjusted	Low	High
Communication				
Simple conversation	High	Adjusted	High	‐
Avoid ‘elderspeak’	Very high	Adjusted	High	‐
One‐step commands and gestures	High	Adjusted	High	‐
Care for clients with a dry mouth				
General treatment				
Appointment with General Practitioner	‐	Additional	High	‐
Additional fluoride				
High‐fluoride toothpaste	High	Adjusted and merged	Very high	‐
Fluoride mouth rinse	Low			
Salivary stimulation				
Lozenges	High	Adjusted	Low	High
Management of symptoms				
Moisture with water	High	Adjusted	High	‐
Salivary substitutes	High	Adjusted	High	‐
Lip balm	‐	Additional	High	‐
Regular preventive oral check‐ups				
Preventive oral check‐ups				
Last appointment	‐	Additional	High	‐
Recall frequency	‐	Additional	High	‐

### 3.1 Preparation phase

The initial draft of the guidelines consisted of 54 items on seven domains: care for natural teeth (13), care for full or partial removable dentures (9), care for the tongue (3), maintenance of oral care utensils (2), approaches for clients who need guidance or help (6), approaches for clients with care‐resistant behaviour (16), and care for clients with a dry mouth (5).

*Survey 1* was completed by 26 participants from 10 countries. The face‐to‐face discussion was conducted with a subgroup of 11 participants from 6 countries. Most participants were dentists, but other professions were represented as well. Twelve items achieved only moderate or low consensus on agreement in *survey 1*. These items were reviewed in the face‐to‐face discussion.

Qualitative feedback contributions included the concern that daily oral care required an individualised, client‐centred approach. In addition, compliance with certain items was questioned due to lack of time and low awareness of the relevance of oral health among nursing assistants. Feedback further revealed that high‐fluoride toothpaste is not available in every country due to heterogeneous pharmaceutical regulations. Markedly diverging opinions were found for the items on chlorhexidine and on chemical cleaning of removable dentures. Feedback also frequently referred to wording and simplification of the items. Participants suggested to include more specific advice for clients with dysphagia. They also recommended additional items such as Sodium Lauryl Sulfate free toothpaste or adaptive aids for toothbrushes. *Preventive Oral Check*‐*Ups* was suggested as an additional domain.

All feedback contributions of the international experts were itemised and critically appraised by the researchers who revised the draft version of the guidelines. The structure of the guidelines was not altered substantially and 13 items remained completely unchanged. An introduction was put in front, one domain and ten items were added, 29 items were adjusted, six items were merged, one item was split and two items were removed. The resulting interim version consisted of 57 items on 8 domains.

### 3.2 Selection phase

Twenty‐seven experts from 12 countries participated in *survey 2*. Very high or high consensus on agreement was gained for 51 items that were included in the final version of the guidelines. In *survey 3*, the remaining 6 items were re‐assessed by 23 participants from 11 countries. The 3 items that gained very high or high consensus on agreement were also included in the final version. Supplementary file 1 shows the final version of the guidelines that consists of 54 items on 8 domains.

### 3.3 Items not included in the final version of the guidelines

Only 3 items of the interim version did not achieve high or very high consensus on agreement. They were not included in the final version of the guidelines. Below, reasons for their inclusion in the draft and concerns of the experts are summarised.

#### 3.3.1 Chlorhexidine application

The interim version of the guidelines advised ‘*Only if brushing is not possible*, *application of chlorhexidine solution with a damp gauze or as a spray can be considered*. *Chlorhexidine can cause adverse effects*. *Consult a dentist or a dental hygienist before use*.’ Advice on chlorhexidine application was found in the Dutch guidelines for long‐term care residents (The Netherlands – Beroepsvereniging van Verpleeghuisartsen en Sociaal Geriaters, 2007). Systematic literature review showed the effectiveness of chlorhexidine on Streptococcus mutans levels (Coelho et al., 2017). High‐quality evidence of a reduction in dental plaque and gingivitis is also available (James et al., 2017). However, the experts in our study raised the concern that nursing assistants might misuse chlorhexidine to substitute daily oral care. Moreover, it was mentioned that brushing is possible as well when chlorhexidine can be applied to the mouth. Participants further emphasised concerns related to accidental swallowing and adverse effects.

#### 3.3.2 Chemical cleaning of removable dentures

The interim version included the advice ‘*After mechanical cleaning*, *the denture can be soaked in water with a denture cleanser tablet*. *It has an additional effect on cleanliness*. *Use denture cleanser tablets only outside the mouth and follow manufacturers’ guidelines strictly*.’ The item was based on the recommendation of international experts who had considered the available evidence (Bartlett et al., 2018). A systematic review confirmed that the combination of mechanical cleaning with chemical agents resulted in optimal denture cleanliness (Papadiochou & Polyzois, 2018). The main concern of the participants in our study was that cleaning tablets might be misused to replace mechanical cleaning. It was further mentioned that clients with cognitive impairment might accidentally drink the cleaning solution. Participants also raised the concern that manufacturers’ guidelines might not be followed, causing damage to denture materials.

#### 3.3.3 Client observes oral care in the mirror

The interim version advised ‘*Clients who resist care by not opening the mouth can be stimulated to open when oral care is provided in front of a mirror*. *The caregiver is standing behind the person reaching around*.’ This strategy was included in a non‐pharmacologic, relationship‐based intervention program that showed to be effective in a randomised clinical trial (Jablonski et al., 2018). Participants in our study mainly raised the concern that it might be non‐ergonomic and uncomfortable for the person who provides oral care.

## 4 DISCUSSION

Recently, an optimised oral health section was developed for the interRAI suite of instruments that is internationally used to assess needs and capacities of care‐dependent individuals (InterRAI, 2020; Krausch‐Hofmann et al., 2020). When clients with insufficient oral hygiene are detected, practice guidelines should be available for nursing assistants who often provide direct hygiene care tasks.

Literature shows that daily oral care is often neglected or only provided superficially in professional care settings (De Visschere et al., 2016; Delwel et al., 2018). Care‐resistant behaviour of clients was identified as one of the main barriers (Göstemeyer et al., 2019; Hoben et al., 2017). Hence, a substantial part of the developed guidelines cover approaches for care‐resistant client behaviour.

Evidence is scarce with regard to optimal daily oral care in older adults. Adoption of study results from younger participants requires caution. Compared to their younger counterparts, older adults often have a higher prevalence of exposed dental roots, larger interdental spaces, and more accumulated tooth loss. In addition, salivation and other oral self‐cleaning mechanisms are affected by reduced mastication activity in clients who ingest pureed food or who receive tube‐feeding. Polypharmacy and senescence itself might further impact susceptibility to oral health disease in older adults (Tonetti et al., 2017).

The design of the current research ensured a stepwise and structured development of the guidelines. In the preparation phase, the available evidence was complemented with practice‐based feedback from international experts. The selection phase made sure that only those items were included in the final version that were supported by broad consensus among the experts.

Online surveys are a practical and efficient method that allows participation regardless of geographical distances. At the same time, effects of peer pressure, social status, seniority, personality and interpersonal dynamics among participants are avoided (Jünger et al., 2012). To also benefit from the advantages of social interaction, a face‐to‐face discussion was conducted with a subgroup of the experts. It has been described that face‐to‐face contact encourages participants to clarify their perspectives and to think about topics more deeply (Krueger & Casey, 2000).

As the interRAI suite of instruments is used internationally, experts from different countries were invited to participate in the study. This approach allowed to take national regulations into account, for example, restricted availability of high‐fluoride toothpaste in Belgium. In addition, contradicting guidelines were harmonised after weighting the different arguments. For example, guidelines from the United States advised overnight storage of removable dentures in water to prevent shrinkage (National Institute of Dental & Craniofacial Research – U.S. National Institutes of Health, 2018). In contrast, dry storage to inhibit bacterial growth is advised in the Netherlands (The Netherlands ‐ Beroepsvereniging van Verpleeghuisartsen en Sociaal Geriaters, 2007). Randomised clinical trials are not available, but an older study reported slight evidence that supports the latter recommendation (Stafford et al., 1986). Hence, in the initial draft of the guidelines, dry storage of removable dentures was advised. In *survey 1*, this item only gained low consensus on agreement. Participants raised concerns related shrinkage of denture materials. The item was considered in the face‐to‐face discussion. Participants mentioned that the water for the dentures was not always renewed daily and that clients with dementia are at risk to drink it. They further argued that perforated denture boxes are available, which might imply that dentures should be stored under dry conditions. Participants suggested that even if the prosthesis is removed from the mouth for a longer period, it can be re‐hydrated before use. They also mentioned that clients who store the denture under dry conditions do not complain when re‐inserting it in the morning. Although the item was not substantially changed, high consensus on agreement was achieved in *survey 2*.

After *survey 3*, consensus on agreement was gained for almost all items (94.7%). However, the participants came from 14 different countries, which is only a part of all nations that are using the interRAI suite of instruments (InterRAI, 2020). Especially, countries from Western Europe and from North America were overrepresented. In addition, different professions relevant to the topic were represented among the experts, but most participants were dentists.

Feedback provided by the experts during the preparation phase alluded the discrepancy between the ideal of an individualised, client‐centred approach and general practice guidelines. However, measures in these guidelines are not mandatory. They rather provide a kit with items for optimal daily oral care. Based on the needs and capacities of the client, relevant parts can be selected when individual care is planned.

Availability of the guidelines will not automatically improve oral hygiene in clients. The guidelines aim to improve knowledge of nursing assistants, but a variety of other aspects also affect the provision of daily oral care, for example, training, time constraints, or clearness of responsibilities (Göstemeyer et al., 2019; Hoben et al., 2017).

## 5 CONCLUSION

Practice guidelines for daily oral care in care‐dependent older adults were developed. The available evidence was complemented with practice‐based feedback and consensus from international experts. The guidelines are now available to complement the optimised oral health section that was recently developed for the interRAI suite of instruments.

## Supporting information

Supplementary Material

## Data Availability

The data that support the findings of this study are available from the corresponding author (JD) upon reasonable request.
