# Supplementary material for: Development of practice guidelines for daily oral care in care‐dependent older adults to complement the InterRAI suite of instruments using a modified Delphi approach
Source: Int J Older People Nurs. 2020 Oct 19;16(1):e12351. doi: 10.1111/opn.12351 (PMC7816227; doi:10.1111/opn.12351)
Supplement: Supplementary file 1 — Supplementary Material [file OPN-16-e12351-s001.docx]

# Introduction

*The guidelines below provide practical advice regarding daily oral hygiene for care-dependent older individuals. If oral hygiene of the client is poor, first observe his/her own approach to determine what kind of help is needed. While for some clients verbal instructions are sufficient, others depend on hands-on assistance. The guidelines are based on scientific evidence to ensure optimal oral care. However, sometimes a modified approach is required due to the condition of the client. An adaptation or learning period might be needed before the guidelines can be fully applied.*

*Please note that the guidelines focus on daily oral hygiene care. They do not cover additional aspects that are as well relevant for oral health such as food counseling.*

# Natural Teeth

*Major dental problems are prevented by regular brushing with fluoride toothpaste and cleaning between teeth.*

- Use a standard toothpaste that contains fluoride.
- Clients who frequently suffer from mouth sores may benefit from using a toothpaste free of the foaming agent Sodium Lauryl Sulfate (SLS).
- A manual or a powered toothbrush can be used. A powered toothbrush based on rotation-oscillation removes plaque more efficiently.
- Use a toothbrush with soft or medium bristles and a small head that permits easy oral access.
- Adaptive aids are available to facilitate dexterity, such as three-headed toothbrushes, foam grips or ergonomic toothbrush handles.
- Brush twice daily: before bed and on one other occasion.
- Make sure that each surface of the teeth, including the gum line, is brushed well.
- Spit out after brushing and do not rinse to maintain fluoride in the mouth.
- Clean between teeth with interdental brushes once a day. Interdental brushes are available in different seizes.
- Clients with obvious caries, dry mouth, or special needs, should receive extra fluoride. Consider a third brushing moment with regular toothpaste, use of high fluoride toothpaste or fluoride mouth rinse. Consult a dentist or dental hygienist for individualized advice and prescription.

# Full or Partial Removable Dentures

*Denture cleaning removes plaque bacteria which is essential to oral and general health.*

- Dentures should be removed from the mouth and rinsed with water after each meal.
- Hold dentures carefully while brushing. Place a clean washcloth in the sink to protect them from breakage if dropped.
- Clean the denture mechanically with a denture brush. Thoroughly remove plaque and remnants of denture adhesive.
- Use a non-abrasive denture cleanser such as liquid soap. Do not use toothpaste as it can damage the denture surface.
- After cleaning, the denture should be stored in a dry and clean box overnight.
- Clients should not keep their dentures in the mouth overnight.
- If clients use denture adhesives, use a gauze to remove remnants in the mouth.

# Denture Retainers in the Mouth

*When the denture is removed from the mouth, fixed retainers can be seen in some clients. The retainers can be based on natural teeth or on implants, they can be detached or connected with a bar. Retainers require the same care as natural teeth.*

- Brush the retainers twice daily with toothbrush and toothpaste.
- Use an interdental brush to clean under the connection bar once a day.

# Tongue

*The tongue is the largest niche for bacteria in the mouth. Cleaning is essential to avoid bad breath.*

- In clients with visible tongue coating, the tongue should be cleaned once a day.
- Use a loop-shaped tongue cleaner.
- To clean, the tongue is extended out of the mouth and stabilized with the free hand. The cleaner is placed as far as possible on the back of the tongue, slightly pressed, and slowly pulled forwards. The scraping movements are repeated several times until the coating is removed.

# Maintenance of Oral Care Utensils

- After use, rinse brushes and tongue cleaner with water and let dry in a clean cup or on a toothbrush rack.
- Replace the toothbrush when the bristles are worn out.

# Guidance or Help with Oral Care

*If guidance or help with oral care is needed, choose an individualized approach depending on the needs and limitations of the client.*

Reminders

- Put pictures in the bathroom showing the different steps of brushing.
- Provide oral care in front of the sink.
- Hand the client the toothbrush to trigger procedural memory.

Hands-on assistance

- Place your hand gently over the person's hand guiding the toothbrush.
- Let the client start brushing and finalize oral care afterwards.

Caregiver provides oral care

- The client should be in an upright position or lie on his/her side to decrease the risk of aspiration.
- Clients who have problems to keep the mouth open, can be helped with a mouth rest or the handle of a second toothbrush placed between upper and lower teeth. It reduces the stress on the jaw joint and on the muscles.
- Use the tell-show-do approach. Tell what will happen, show it, and than do the oral care as you have explained.
- In clients with dysphagia, remove the fluid from the mouth after brushing with a gauze or with a suction device.

# Care-Resistant Behavior

*Daily oral care requires consent. If clients are cognitively impaired, consent needs to be given by their guardian. A variety of threat-reducing techniques is available to manage care-resistant behavior in clients with dementia. Caregivers are expected to select strategies on a trial and error basis.*

Cognitively competent clients

- Establish rapport, explore the reasons for the refusal and explain why oral care is necessary. It ensures that clients can make an informed decision.
- If the client refuses to cooperate even when well informed, this needs to be respected. Resume the discussion at a later point of time.

Clients with dementia

- Have a dental care routine. Same technique and utensils at the same time and place.
- Provide daily oral care in a quiet environment with a minimum of persons present.
- Approach the person at eye level and within her or his visual field.
- Establish rapport by engaging in an affirming and simple conversation.
- Use polite one-step commands, gestures and pantomiming.
- Avoid “elderspeak” (high pitch, sing-song cadence, patronizing tone, collective pronouns, and infantilizing terms).
- Smile and use humor when interacting with the client. Singing also contributes to create a safe and affirmative atmosphere.
- Distract the client by talking or have him/her hold the same item being used in mouth.
- Place your hand over the hand of the client guiding the movements or hand the client the toothbrush to initiate brushing.
- Use gentle touch judiciously to reassure the client and reduce anxiety.
- When care-resistant behavior is escalating, the involved caregiver might be perceived as threatening. Another caregiver can try to take over as clients are more willing to cooperate after being “rescued” by the safe second caregiver.

# Dry Mouth

*Saliva is necessary for a variety of oral functions such as taste, chewing or swallowing. As saliva is also crucial to prevent tooth decay, daily oral care requires particular attention in clients with a dry mouth.*

- Make an appointment with a physician to diagnose causes of the dry mouth and to decide about general treatment.
- Provide extra fluoride for natural teeth. Consider a third brushing moment with regular toothpaste, use of high fluoride toothpaste or fluoride mouth rinse. Consult a dentist or a dental hygienist for individualized advice and prescription.
- Salivary substitutes applied as a gel, rinse or spray are helpful to reduce discomfort.
- Clients should sip water frequently to ensure hydration and to moisture the mouth. Water spray or thin iced water chips can be used for clients with dysphagia.
- Lozenges are tablets that dissolve slowly in the mouth. They can stimulate salivary flow, but should be free of acid and sugar and only be offered to clients who do not suffer from dysphagia.
- Apply a lubricating lip balm on dry and chapped lips.

# Regular Preventive Oral Check-Ups

*Regular check-ups by a dentist or by a dental hygienist prevent (further) decline of oral health. Natural teeth, gums and functionality and fitting of the denture are examined.*

- Ask clients or family members when the last preventive oral check-up was done. If this was longer than six month ago, suggest to arrange an appointment.
- The oral health professional decides about recall frequency.
